# Potentially Critical Driving Situations During “Blue-light” Driving: A Video Analysis

**DOI:** 10.5811/westjem.2022.8.56114

**Published:** 2023-01-03

**Authors:** Maria J. Prohn, Britta Herbig

**Affiliations:** Institute and Clinic for Occupational, Social and Environmental Medicine, University Hospital, Munich, Germany

## Abstract

**Introduction:**

Driving with warning lights and sirens is highly demanding for ambulance drivers, and the crash risk is much higher than that during normal driving. In this study our goals were to establish a coding protocol to observe how often and how long potentially critical driving situations (PCDS) occur during “blue-light” driving (driving with emergency response lights) and to describe traffic and environmental conditions preceding and accompanying the PCDS.

**Methods:**

We collected randomly drawn video data of real ambulance driving between 2014–2017 in two German federal states. A coding protocol was developed to categorize PCDS into four types (“right of way,” “crosswalks,” “overtaking” [passing], and “other”) and to describe them within the context of road characteristics, incident type, traffic, weather conditions, and driving style.

**Results:**

A total of 172 videos of 71 different drivers were chosen randomly covering 1125 minutes of driving with warning lights and sirens. The drivers had a mean age of 33.7 years, and 25.4% were female. A total of 2048 PCDS occurred with a mean duration of five seconds (range of 1–66), amounting to one PCDS every 33 seconds. Twenty percent of the driving time involved PCDS. The rapid driving style (10.5%) showed more PCDS (one every 28.5 seconds), and the defensive driving style showed fewer PCDS (one every 49.6 seconds). Of all detected PCDS, “right of way” situations (57.5%) were most frequent, followed by “overtaking” [passing] maneuvers (30.2%).

**Conclusion:**

This study used a detailed coding protocol to describe driving with warning lights and sirens. The PCDS occurred less frequently than anticipated, although they were still common events when driving an ambulance, representing significant potential for crashes or near-crashes. These results can be used for insight training programs to raise ambulance drivers’ awareness of typical PCDS and associated potential crash risk.

## INTRODUCTION

Driving emergency vehicles with warning lights and sirens has a much higher crash rate than normal driving. In Germany, the rates were found to be fourfold for fatal crashes, eightfold for serious injuries, and seventeenfold for material damage.^1^,^2^ In other countries, the crash rates for emergency vehicles have been reported as three to five times higher than non-emergency driving.^3^,^4^ Emergency vehicle crashes not only affect directly involved persons but also result in delayed help at the actual emergency scene and in the broader community. They further involve more people with injuries compared to crashes of similar-sized vehicles.^5^,^6^

Emergency vehicle crashes are often caused by their drivers^1^ and occur most often at intersections and in overtaking [passing] situations.^1^–^7^ Previous studies have reported critical driving situations occurring every 19 seconds during emergency lights usage (“blue-light” driving).^1^,^2^ Critical driving situations have been defined as situations that involve risky driving behavior by emergency vehicle drivers,^2^ by other road users,^8^ or regarding perception time for evasive maneuvers.^9^ Operationalization often includes crashes or near crashes^9^ as well as driving triggers.^10^–^12^ The analyses of near crashes in naturalistic driving studies can be evaluated with triggers from accident recording systems,^10^–^12^,^13^,^14^,^15^ and video analyses of drivers’ reactions to incidents or distracting behaviors.^16^,^17^ The use of front cameras allows the analysis of normal driving from the drivers’ perspective.

To our knowledge, the only available video data for critical driving situations during emergency driving with warning lights and sirens was reported in 1972.^2^ Since then, a number of technical (eg, driver assistance systems or vehicle construction), organizational (eg, training possibilities), and traffic (eg, increasing volume) conditions have changed. In this study we aimed to provide up-to-date data on potentially critical driving situations (PCDS). Our first objective was to establish a coding protocol to analyze video data of real emergency driving for PCDS regardless of whether they resulted in hazards or increased crash risk. We define PCDS as driving situations where ambulance drivers need heightened attention due to violating traffic regulations (eg, running a red light), stretching traffic regulations (eg, overtaking another vehicle in uncommon situations), and driving differently than normal (eg, crossing road markings to have more space), or when reactions due to traffic conditions are necessary. Against this background, the second objective was to describe the type and frequency of PCDS during emergency driving as well as traffic and environmental conditions preceding and accompanying the PCDS.

The results of this study are intended primarily for research on accident causes and risks associated with blue-light driving. Subsequently, they can be used in training courses for blue-light drivers to adapt their driving behavior. In addition, they can help co-drivers to have a positive influence on driving behavior. Emergency physicians would benefit from these changes as it is in the interest of all involved to make blue-light driving as safe as possible.

## METHODS

### Data Collection

Driving data of paramedics from different rescue services during regular work shifts were recorded in the context of a training evaluation study between October 2014–June 2017 in two German federal states.^18^ The Ethics Committee at the Faculty of Medicine of Ludwig-Maximilians-University Munich approved the study (ID: 206–14), and all participants gave written informed consent. Data collection took place in rural and urban areas at all times of day. Two different ambulance vehicle types were included: rescue transport vehicles (“RTV,” a light truck of approximately 4.7 tonnes, staffed with at least one emergency medical technician (EMT) as driver and one paramedic as co-driver) and emergency physicians’ response vehicles (“NEF,” usually a car less than 3.5 tonnes, staffed with one EMT or paramedic as driver and an emergency physician. Both vehicle types typically respond to emergencies. While the RTV is responsible for transporting patients to the hospital, the NEF brings the emergency physician and necessary equipment to the scene (so-called “rendezvous system”). The vehicles were equipped with cameras to record traffic in front of them, without audio recording. Recording started when the ignition of the vehicle was turned on and ended 10 seconds after it was turned off.

**Population Health Research Capsule**

What do we already know about this issue?

*Crash risk is higher in emergency driving. The characteristics of potentially critical driving situations (PCDS) that can cause accidents are still unclear*.

What was the research question?

*Our goal was to develop a coding protocol and to quantify type and frequency of PCDS during real-life emergency driving*.

What was the major finding of the study?

*Every 33 seconds a PCDS of about 5 seconds occurs during driving with warnings lights and sirens; 57.5% are right-of-way situations*.

How does this improve population health?

*Knowing potentially critical situations during emergency driving may improve driver training, reduce accident rates, and ensure rapid assistance in emergencies*.

### Video Data

For this study, we used video data of driving with lights and sirens to an emergency scene to identify PCDS. All driving of an emergency vehicle by the participants in several work shifts was recorded. As several operations did not include driving with warning lights and sirens, we included 1–4 emergency driving videos of sufficient length for each participant. Sufficient length was defined as a duration of 4–10 minutes. The minimum and maximum were set according to the mean of all driving times to an emergency scene (M = 6:04 minutes [min]; SD = 3:34 min) to avoid unrepresentative drives. The upper limit corresponds to the mean plus SD, rounded up. The same calculation for the lower limit would have been at about 2.5 min, assuming per the literature^1^–^2^ that a critical event should occur approximately every 19 seconds; however, only 7–8 events would occur in 2.5 min and, thus, significantly fewer than in the longer videos. Therefore, it was decided to raise the lower limit to 4 min.

We included the videos if they met the following criteria: 1) the videos showed a drive to an emergency scene with lights and sirens with 2) a driving time between 4–10 minutes. If no video with a driving time of sufficient length was available, two shorter (third step) videos were randomly drawn. Only when the recordings from a participant did not contain one video with a sufficient length or two shorter ones, one longer video was selected (fourth step). All drawings were made randomly via the numbered list of videos and the random function of Excel (Microsoft Corporation, Redmond, WA).

A total of 4,487 videos with 27,356 minutes of recordings met the first inclusion criterion (drive to an emergency scene). Of these videos 2,749 with 17,330 minutes of recordings met the second inclusion criterion (driving time between 4–10 minutes). Along with stratification for the driver, 172 videos totaling 1,125 minutes from 71 participants were drawn randomly. The characteristics of these drivers are presented in Table 1. This table indicates that the included video data capture different driving environments and experienced as well as inexperienced drivers.

### Coding Protocol

To identify PCDS in the video recordings, we used categorization based on a coding protocol. As described above, PCDS were defined as driving situations where ambulance drivers need heightened attention due to violating traffic regulations (e.g., running a red light), stretching traffic regulations (such as overtaking another vehicle in uncommon situations), and driving differently than normal (eg, crossing road markings to have more space), or when a reaction due to traffic is necessary. To develop the coding protocol the existing protocol was adapted and extended.^2^ In that it was differentiated between critical driving situations (pulling into moving traffic, jumping red traffic lights, intersections without traffic lights, as well as overtaking in traffic jams, standing traffic in front of red lights, on straight roads, in bends, on road gradients or on two-lane streets), wrong reactions of ambulance drivers (overtaking on the right, driving the wrong way, completely using the oncoming lane for overtaking, impeding other drivers in forming a corridor for the emergency vehicle [“*rettungsgasse*,” a specific German term similar to the American or Canadian “move over” laws] by signaling the wrong direction, jumping red lights without adequate deceleration and disregarding the right of way without deceleration), and wrong reactions of other road users (sudden braking, accelerating before the ambulance, accelerating before sudden braking, hindering to form a corridor for the emergency vehicle, and no swerving to give way).^2^ The authors used 54 minutes of driving data to analyze critical driving situations.

For the development of our coding protocol, a total of 212 minutes of recordings were rated by three observers, one of whom was a study author. During development, the protocol was discussed and adapted iteratively to increase its clarity and interrater agreement. For this iterative process there were two sets of videos, 10 (85 min) in the first set and another 20 (127 min) in the second set. Usually, two of the three observers viewed a portion of the video sets, discussed ratings, and refined the protocol. Therefore, videos were sometimes rated multiple times when the protocol was changed in relevant points.

The differences between the final protocol and the older coding protocol^2^ are as follows: the false reactions of other road users are not coded; and our categories (types) involve all critical driving situations except for overtaking on road gradients. Additionally, other driving situations are coded (eg, yellow/green lights, roundabouts, pedestrians, other vehicles with warning lights and sirens, and animals). All PCDS were coded in more detail and with better comparability concerning road class, other traffic (traffic density, cross and oncoming traffic), number of lanes, duration, reaction, and road, weather, and lightning conditions. For each video, the driving style was coded. The final protocol with detailed information on the coding and examples can be found in Supplemental Files A (German version) and B (English version).

Each video was first searched for primary observation units (“types”): “right-of-way” situations, “crosswalks,” “overtaking” maneuvers, and “other” situations. Subsequently, for every type, the following subcategories were coded for each incident if relevant: *road class; incident type; size, traffic density; cross traffic; oncoming traffic;* and *traffic in driving direction*. We further coded *road, weather and lighting*, as well as *reaction to incident*, for each PCDS. The *reactions to incident* was the only subcategory with multiple coding. Seven different reactions (no reaction/consistent driving; swerving; braking without stopping; stopping/braking to a halt; accelerating; stopping acceleration; and turning/using an alternate route) are included in the protocol and could be combined to describe these reaction in detail. For example, the emergency driver could only brake or both brake and swerve due to a PCDS. All reaction descriptions could be combined except for the “no reaction” code.

**Figure 1:**
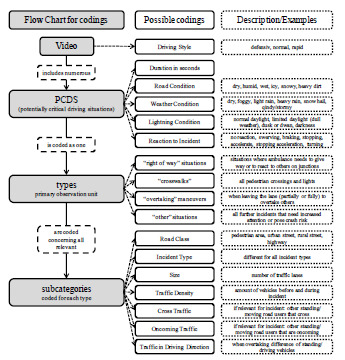
Flow chart of the coding process.

Additionally, for all incidents, the *duration* was coded, and raters could give a qualitative comment to provide context for the incident. For each video, the *driving style* was subjectively coded for each driver on a three-point scale (1 - defensive, 2 - normal, and 3 - rapid). In each case we performed the coding of the driving styles after the entire video was evaluated. Subjective assessment of the driving style was based on accelerating after intersections, keeping distances, exceeding speed limits, and sharp steering behavior. A defensive driving style was characterized by steady and predictable driving. A rapid driving style was characterized by reduced following distances, speeding, and abrupt steering maneuvers. Figure 1 shows an overview of the coding process.

The last step before finalization of the coding protocol was a test with the same three raters using nine randomly drawn videos covering 62 minutes of driving. The interrater reliability of type classification using Fleiss kappa was 0.785 when all three raters coded a PCDS. A total of 54.4% of the PCDS were detected by all three raters; another 19.8% were detected by two raters. The missing detection was often due to the length of the incidents. For overtaking maneuvers, for example, it was possible to code one long incident or two or more shorter ones. To improve the reliability of classification we added instructions on when to start a new incident to the coding protocol. After final adjustments of the coding system a fourth rater was instructed to use the protocol. Training was performed with already coded video recordings until the fourth rater was able to use the coding protocol as reliably as possible. Video recordings used for the development of the coding protocol were not included in the later analyses since changes in the protocol were not compatible with the coding of older protocol versions.

### Statistical Analyses

Although the main goal of this study, in addition to the development of a coding protocol, was to provide an overview of the frequency of the pertinent details of PCDS and percentages and counts, we also provide χ^2^ statistics from crosstabs for most categories and characteristics to give an impression of whether certain aspects differ significantly. As χ^2^ statistics are difficult to interpret for crosstabs with more than two categories, χ^2^ statistics for reduced crosstabs (combining all categories not in the focus of the analyses into one) are also presented. For effect sizes, phi (for 2 × 2 crosstabs) or Cramer’s V (for crosstabs containing more categories) was used with the following rule of thumb for evaluation: below .20 = weak association (small effects); .20–.40 = moderate association (medium effects); and .40–.60 = relatively strong association (large effects).^19^

## RESULTS

### Interrater Reliability of the Final Coding

After the fourth rater evaluated all videos included in the final analysis, approximately 10% (15 videos, 108 out of a total of 1,125 minutes) were rated by a second observer (author) to ensure interrater reliability of the final coding protocol. Agreement in detected PCDS was 88.2%. The cumulative duration of 224 detected PCDS of the fourth rater was 25:08 minutes. The other rater detected 236 incidents with a cumulative duration of 24:34 minutes. A total of 29 PCDS (11.8%) were detected by only one of the raters. Of these, 16 (55.2%) were exclusively due to different lengths of the PCDS, an additional five (17.2%) were rated only by the fourth rater, and eight (27.6%) were rated only by the second observer. For the interrater reliability analyses, we used the 216 PCDS that both raters detected (see Table 2). In addition, only identically coded types were used for subcategories whose coding depends on the type (incident type, traffic density, cross traffic, oncoming traffic, traffic in driving direction). Driving style was coded once for each video.

Interrater reliability for most codes was very good (most intraclass correlation coefficients [ICC] show good to excellent agreement,^20^ most Cohen’s kappa coefficients almost perfect agreement^21^). Only three variables show lower interrater reliability: oncoming traffic (moderate ICC^20^) as well as reaction to incident and traffic in driving direction (both substantial kappa).^21^ The moderate interrater reliability for oncoming traffic and traffic in driving direction was mostly attributable to the assessment of traffic as moving or stopping.

### Overall Characteristics of PCDS

During 1,125 minutes of recorded driving with blue light and sirens, 2,048 PCDS occurred (one PCDS every 33.0 seconds). The mean duration of blue-light driving was 6.5 (range = 2–11) minutes. The median duration of PCDS was 5 seconds (mean = 6.6, range = 1–66) with no significant difference between RTV and NEF vehicles (T = 0.248, *P* = .81). Overall, 20% of the driving time involved PCDS. In 932.5 recorded minutes of RTV driving, 1,663 PCDS occurred (one PCDS every 33.6 seconds). For NEF, PCDS occurred more frequently: 385 PCDS in 192.5 recorded minutes (one every 30 seconds). The differences between vehicle type and PCDS in the time frames are significant but with a very low effect size (χ^2^=6.0, *P* = .01, ϕ =. 010); that is, there was probably no effective difference between both vehicle types.

For most of the video recordings, a normal driving style was coded (82.0%). A total of 7.6% and 10.5% of the drivers showed a defensive or rapid driving style, respectively. The driving style was associated with the rate of PCDS occurrence. For the normal driving style, almost the same number of PCDS as the overall number was coded (one every 33.0 seconds). A defensive style was associated with a lower PCDS rate (one every 49.6 seconds), whereas a rapid driving style had a higher rate (one every 28.5 seconds).

**Figure 2:**
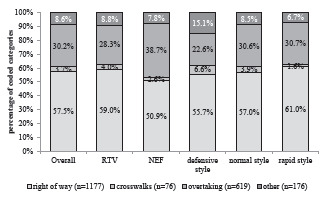
Overview of the PCDS types for the overall data as well as by vehicle type and driving style. *PCDS*, potentially critical driving situations.

The most frequent type of PCDS involved “right-of-way” situations (57.5%), followed by “overtaking” maneuvers (30.2%), “other” situations (8.6%). and “crosswalks” (3.7%). For RTV, the number of “right-of-way” situations was higher than for that of NEF (59.0% vs 50.9%), and for “overtaking” maneuvers, it was reversed (RTV 28.3% vs NEF 38.7%). Although this difference is significant, the effect size is very small (χ^2^=16.8, *P* = .001, Cramer’s V = .090). For the different driving styles, there was almost no difference between normal and rapid driving (χ^2^ = 14.7, *P* = .02, Cramer’s V = .060); however, a defensive driving style was associated with a higher percentage of “other” situations (15.1%), and “crosswalks” (6.6%) but fewer “overtaking” maneuvers (22.6%) (see Figure 2).

The environmental conditions were comparable for all four PCDS types (see Table 3). The streets and weather were mostly dry. Light rain and, therefore, damp streets as well as heavy rain and wet streets were also rather common. More extreme weather and ground conditions were not observed in the analyzed PCDS situations. As they were present in the recordings used to develop the protocol, they are included for the sake of completeness. Approximately half of the incidents occurred during normal daylight. Some small differences can be seen in “overtaking” maneuvers where heavy rain and wet streets more often occur compared to the other PCDS. This might also explain the higher number of limited daylight (dull weather) in “overtaking” maneuvers. Darkness was found more often in “right-of-way” and “crosswalk” situations.

Most PCDS occurred on urban streets; only approximately 10% were on rural streets or highways. At first glance, this seems comparable to the number of participants who work in rural areas. However, the observed 10% PCDS on rural streets happened in approximately one third of all videos, not just in videos of participants located in rural areas. Fourteen of the videos with rural streets (23.3%) stem from participants located in rural areas, 27 videos (45%) stem from participants located in suburban areas, and another 19 videos (31.7%) stem from participants located in urban areas. For the videos where all PCDS occurred just on urban streets, most participants’ emergency rescue services were defined as being urban (69.6%) or suburban (25.9%); however, rural ones also occurred (4.5%). This shows that strict differentiation is not possible between regions of driving concerning potentially critical driving situations.

### Characteristics of “Right of Way” Situations

Overall, 1,177 “right of way” incidents (57.5%) were coded with a mean duration of 6.4 seconds (range = 1–35). Concerning road class, most “right of way” situations occurred on urban streets (92.1%), followed by rural streets (6.5%), pedestrian areas (0.2%) and highway ramps (1.2%). The number of lanes (size) was mostly one (63.6%), followed by two (23.9%) or more than two lanes (12.6%). The majority of incident types were red lights (38.7%), junctions without signs (30.1%), and stop/yield signs (18%), followed by roundabouts (8.1%), right of way (3.1%), and yellow lights (2.1%).

The traffic density during “right-of-way” incidents was mostly quite low, which could be due to the number of lanes, the kind of street (small side streets or rural areas), or the reaction of other road users that gave the ambulance a free lane. In 77.4% of the cases, at least one lane was clear to pass other vehicles or no vehicles at all were in front of the intersections. In 20.5% of the cases, the ambulance had no problems passing either a few vehicles (16.9%) or heavy traffic (3.6%). In 2.1% of the cases, the ambulance was obstructed by either a few vehicles (0.9%) or heavy traffic (1.2%). In situations with oncoming traffic (when turning left or using most of the oncoming traffic lane) or cross traffic, there were no other road users the driver needed to pay attention to in 58.1% of the cases. However, in 19% of the cases, there were stopping/standing road users; in 14.4% at least one road user was initially moving before letting the ambulance pass; and in 8.5% of the “right-of-way” incidents at least one road user did not notice the ambulance and did not let it pass.

### Characteristics of “Crosswalk” Situations

“Crosswalk” situations made up a very small number of the PCDS (76; 3.7%) and had an average duration of 2.7 seconds (range = 1–9). However, in this incident type, the most vulnerable road users were pedestrians and cyclists. Most incidents occurred on urban streets (98.7%); only one incident was on a rural street (road class). In one “crosswalk” situation, the driver had more than one lane to choose from (size). Most “crosswalk” incidents (incident types) were pedestrian crossings (92.1%), followed by red pedestrian lights (6.6%) and green pedestrian lights (1.3%). Yellow pedestrian lights did not occur during the observations.

Concerning cross traffic, there were mostly no pedestrians (84.2%), or few pedestrians who gave way to the ambulance (15.8%). In 92.1% of the cases, no vehicles were in the lane before the “crosswalk” situation, followed by 6.6% of the cases, with few vehicles and no problems of passing (traffic density). In one case (1.3%), at least one vehicle obstructed the ambulance in front of a pedestrian crossing. For oncoming traffic, stopping drivers, initially driving and then stopping road users, and at least one driver not noticing the ambulance were each coded once (1.3%) in the “crosswalk” situations.

### Characteristics of “Overtaking” Maneuvers

The 619 (30%) coded “overtaking” maneuvers were on average 5.9 sec long (range = 1–38). Compared to the other types, more incidents occurred on the road class of rural streets (15.2%) and on highways (2.3%). Nevertheless, the majority of “overtaking” maneuvers occurred on urban streets (82.4%). In most “overtaking” situations one lane (85.3%) was available, followed by two lanes (13.1%) or more than two lanes (1.6%) (size).

**Figure 3:**
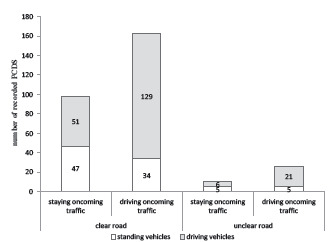
Extraction of overtaking maneuvers (for full data see Supplemental File C): representing oncoming traffic (bars) and traffic in driving direction (legend) depending on the clarity of the road (horizontal axis). *PCDS*, potentially critical driving situations.

Concerning incident types, the situation was mostly clear (74% straight roads and 14.5% clear bends), so oncoming traffic could be evaluated by the drivers. Traffic jams occurred very rarely (0.2%). However, in 11.3% of the maneuvers, the drivers started the overtaking maneuver even though the street was obscured; therefore, the oncoming traffic could not be evaluated appropriately (4.0% unclear straight roads and 7.3% unclear bends). Most “overtaking” maneuvers had no oncoming traffic (42.6%), followed by driving (30.5%) and standing (17.6%) oncoming traffic. Figure 3 displays some situations of oncoming traffic and traffic in the driving direction by incident type (see Supplemental File C for an overview of all overtaking combinations).

Constructional separation existed in 5.8% of the “overtaking” maneuvers. Overtaking in the corridor for the emergency vehicle (1.9%) or on the right-hand side (1.5%) was very rare. Mostly 1–3 vehicles (traffic density) were overtaken (86.1%), followed by small convoys with up to nine vehicles (12.0%). Larger convoys were overtaken in 1.9% of the maneuvers. The traffic in the driving direction was 67.5% driving and 32.5% standing. Overtaking situations on unclear roads with many driving vehicles that need to be overtaken (large convoys) and driving in oncoming traffic are likely the most hazardous situations. Such situations did not occur in the coded videos. However, 0.2% of the overtaking situations were coded as overtaking of small driving convoys when traffic in the driving direction was moving in unclear bends. In one quarter (24.2%) of all “overtaking” maneuvers, traffic in the driving direction and oncoming traffic were moving.

### Characteristics of “Other” Situations

A total of 176 (8.6%) incidents were coded as “other” situations with less precisely operationalized subcategories. On average, with a duration of 11.4 seconds (range = 1–66), the situations were longer than the other types. The road class was comparable to the first two types (94.9% urban, 4% rural). However, “other” situations occurred more often on pedestrian streets (1.1%) and primarily where there was one lane (90.9%) followed by two lanes (9.1%).

Obstruction (27.3%) was the most frequent incident type within “other” situations, followed by driving the wrong way (21.6%), turning or losing the way (18.2%), and driving on a narrow road (16.5%). Lane change to specialized lanes, turfs or walkways made up 2.8% of the “other” situations. The remaining 13.6% contained other incidents, such as running children or cyclists on the street, barriers on the street, or stopping to let someone get on board. The traffic density categorization within the “other” situations showed more blocked roads than in the other types.

### Characteristics of Reactions to Incidents of All Four PCDS Types

Table 4 gives an overview of the different reactions of ambulance drivers to PCDS types by driving style. Often, the drivers showed no reaction to PCDS (see Supplemental File C). Especially in crosswalk situations, incidents without reactions outnumber those with reactions. The chi-square test used to compare PDCS types and reactions shows a significant medium-sized effect (χ^2^=225.0, *P* < .001, Cramer’s V = .331). In 17.1% of the coded PCDS the ambulance driver showed no reaction.

This strikingly high number of no reactions in crosswalk situations (63.2%) might be due to the high number of no vehicles in front of crosswalks (92.1%) and no pedestrians on them (84.2%). In four situations with no reaction, there were a few pedestrians that let the ambulance pass; in all other situations there were no other road users. The differences between driving styles and reactions in the “crosswalk” situations are not significant.

The high number of no reactions in the “right-of-way” situations (22.5%) might be explained (at least in part) by the fact that often no or few other road users obstructed the ambulance. However, when looking at the other subcategories combined, in four cases there was moving cross traffic but no reaction to it. At 27 red lights (10.1%), 56 stop/yield signs (21.1%), and 138 intersections without a sign (52.0%), the ambulance driver continued to drive as before. These PCDS might rapidly change into critical situations or even (near) crashes. The rapid driving style showed a higher number of no reactions and swerving but a lower number of braking behaviors compared to the other two driving styles.

For the “overtaking” maneuvers in PCDS, a much lower number of no reactions were found, which was due to the higher number of swerving with or without braking. These reactions show significant differences with small- to medium-effect sizes between the different driving styles, namely, more swerving for drivers with a rapid driving style and more serving combined with braking for normal driving style.

The reactions to “other” situations vary much more due to the mix of situations that are summed up in this type. No reaction to “other” situations was mostly present in the rapid driving style, followed by the normal driving style. In one of those situations, vehicles were in front of the ambulance, and in three other cases, there were initially driving/moving road users in crossing or oncoming traffic that let the ambulance pass. These cases might have easily ended in more critical driving situations if the other road users had not reacted as properly as they did. The defensive driving style shows a high number of a mix of different reactions to the PCDS. This effect has a medium-effect size.

## DISCUSSION

In this study we aimed to establish an objective protocol for the video analysis of emergency driving situations and to describe these situations in detail. We successfully developed a detailed and extensive coding protocol with good-to-excellent interrater reliability for most assessed codes and analyzed a large amount (1,125 minutes) of driving with blue light and sirens in actual traffic in urban and rural areas. Moreover, as 71 drivers from different parts of Germany and different rescue services with a wide range of working and driving experience were included, our data provides a broad picture of traffic safety while driving with emergency light and sirens. They show how often drivers need to pay greater attention to traffic due to the necessity of stretching or disregarding traffic regulations, or to react to traffic in some other way.

Overall, 2,048 PCDS occurred, that is, one PCDS every 33 seconds of driving with blue light and sirens. This is less frequent than that previously found but is still a very common event. Twenty percent of the driving time involved PCDS. During an average blue-light run of approximately seven minutes, the driver had to deal with more than 12 PCDS. This is a much higher number of potentially critical incidents than “real” incidents found in general by other researchers who used triggers and detected one incident every 350 kilometers or every five hours.^12^

We showed the high potential for critical situations and crashes. The PCDS found in this study can easily precede crashes or near-crashes if minor circumstances change, such as one car of an overtaken convoy pulling out or a road user not noticing the ambulance. In line with this reasoning, more than half of the PCDS occurred during “right-of-way” situations where the ambulance driver mostly needed to deal with a red light, or an intersection without any signs or a stop/yield sign. Another 30% of the PCDS were overtaking maneuvers. Intersection and overtaking events are situations with the highest crash risk for driving with blue light and sirens.^3^,^4^,^6^ Crosswalk-related PCDS were rather rare; however, they bear a high risk for the most vulnerable groups in traffic – pedestrians and in particular children – who cannot evaluate vehicles’ speed.

Road users in general were considered in three subcategories, namely, traffic density, crossing and oncoming traffic, and, for overtaking maneuvers, traffic in the driving direction. In most PCDS, there were no or few other road users involved, and these mostly did not hinder the ambulance. The reasons for this might be the number of lanes, allowing for at least one free lane when other road users reacted correctly to the ambulance. Additionally, specialized lanes often lead to a free space to drive through an intersection. A blocked road or a road user continuing to drive/walk without letting the ambulance pass (8.6% of the cases) does not necessarily mean that the respective drivers did not react correctly: it could also be that there was no space to get out of the way.

This data suggests that most road users acted correctly or at least attempted to cede the right of way to the ambulance as they were supposed to do. One reason for the correct reaction of other road users might have been an early start of the blue light and siren by the ambulance drivers (which was not recorded). The earlier the signals are activated, the more time other road users have to free the way and, even more importantly, orient themselves in the situation to cede the right of way in a controlled manner without endangering themselves or other road users. However, these are assumptions from the rather small number of coded involvement of others even on urban streets, as other road users were not directly observed. Nevertheless, this is a particularly important point concerning the training of ambulance drivers. This suggests not only that other road users often at least try to act correctly and give way to the ambulance but also shows the potential high impact of the ambulance drivers’ correct (or incorrect) behavior in the situation.

Against this background, we also analyzed the reactions of emergency vehicle drivers to PCDS. Most often, the drivers braked and/or swerved due to the PCDS. However, in more than one fifth of the “right-of-way” situations and even two thirds of the “crosswalk” situations, the driver did not react to the PCDS at all and continued driving as before. In those situations, a crash can easily happen due to a misinterpretation of the behavior by other road users or inattentiveness due to other distracting tasks, such as thinking of the upcoming operation or using radio communication. Evasive maneuvers have been found to play a role in crash prevention, which shows the importance of reacting correctly to PCDS.^14^

Independent of the behavior prior to incidents, such as correct use of directional signaling, early use of warning lights and sirens or adequate speed and distance, the reaction to an incident is important to address in driver training classes. Each swerving requires space that needs to be considered. Joint braking and swerving produces high forces on the ambulance vehicle. No reaction at all can easily lead to critical driving situations. Crashes without reactions beforehand might end up with legal consequences. However, reactions that might confuse other road users, such as stopping at intersections despite other road users having noticed the ambulance, might also lead to uncontrolled reactions of others and to critical situations. Thus, the reactions to incidents while driving with warning lights and sirens should be part of practical or at least simulated training of ambulance drivers.

Finally, the driving style itself plays a role. For the rapid driving style, a higher number of PCDS was found, and for “right-of-way” and “other” situations, drivers with a rapid driving style often showed significantly more lack of reaction to the PCDS. In “overtaking” maneuvers, ambulance drivers with a rapid driving style more often reacted by swerving without braking, whereas those with normal driving style more often reacted with swerving and braking together. The defensive driving style was shown in “other” situations, often a mix of different reactions. Although it makes sense that a more rapid driving style would lead to more PCDS per minute and a more defensive driving style would lead to a lower rate, the reverse could also be true: the raters might have given their general impression of the driving style based on the number of PCDS they observed.

## LIMITATIONS

In addition to this potential confounding matter of how raters may have judged the driving style of emergency responders, our study has additional limitations that should be considered. Information on the initial speed when assessing the reaction to the PCDS was missing, and other road users were not coded. Although the coding protocol was developed with several raters, just one rater observed all videos. To reduce possible systematic coding mistakes, this rater was instructed in detail, some videos were double-checked, and difficult situations were discussed. The agreement on detected incidents found in the final interrater reliability still has potential for improvement. The relatively lower agreement for the codes “oncoming traffic” and “traffic in driving direction” could have different reasons. One is that it was often difficult to determine whether vehicles were stopping or driving on the video especially if the other drivers recognized the ambulance and decelerated. If they were almost stopped it could be that raters differently assessed this situation. Another reason could be the different lengths of the incidents, especially the “overtaking” maneuvers.

However, the rater who had the lower number of detected incidents rated all videos; this suggests an underestimation of actual PCDS. The effort required to evaluate the videos is large (up to eight times the video time), so only a smaller portion of videos, but a substantially larger amount than in previous research, was analyzed. Therefore, it cannot be excluded that the analysis of more videos would change the results. However, due to the random selection of the evaluated videos and the wide range of included drivers, we are confident that the key messages would not change. It would be an interesting addition to use artificial intelligence to enable automated video evaluation for at least parts of the observation protocol to be able to evaluate a much larger number of videos.

The sample consists of volunteer participants who knew they were part of a study and might, therefore, have driven less dangerously or aggressively. However, the participants were measured numerous times, which might have led to habituation to the situation of being observed, and the analyzed videos were drawn randomly. Moreover, a rather large sample in terms of analyzed time and drivers of various backgrounds participated, so we are cautiously confident that the data is generalizable to regions with comparable traffic and legal regulations for driving with warning lights and sirens. However, it must be considered that regulations for training programs vary between countries even within the European Union.^22^ Nevertheless, as we included experienced and inexperienced drivers with different licenses, and different working and driving experience the general direction of the data will likely hold true.

## CONCLUSION

This study presents a unique overview of potentially critical driving situations while driving with emergency lights. The risk of a PCDS evolving into a critical situation or crash is high but has not been quantified. Although they occur less often than previously reported, PCDS still make up 20% of the driving time. Typical PCDS situations as well as those that are less frequent but pose a high risk can be used for educational programs. Ambulance drivers should become more aware of those typical – usually not interpreted as risky – situations and learn how to manage them to increase the traffic safety of emergency response driving. Although a number of PCDS are dependent on other road users’ reactions, ambulance drivers continue to have the highest impact on traffic safety while driving with warning lights and sirens. Traffic safety training should, therefore, be the content of education and training of all emergency medical personnel driving ambulances. The PCDS and issues found in this study can be used as examples and starting points in such trainings to raise awareness for critical situations and their common occurrence, and to discuss and train for their prevention and adequate responses. This might help to support a mutual understanding between ambulance drivers and other road users.

## Supplementary Information







## Figures and Tables

**Table 1:** Characteristics of the included drivers.

	N	Mean	SD	Range
Age (years)	71	33.7	9.5	20 to 65
Working experience (months)	70	127.8	103.4	12 to 456
Average monthly driving time of ambulance vehicle (hours)	66	88.0	54.2	8 to 192
Driving license possession (years)	71	14.9	8.8	2 to 44
Gender				
18 (25.4%) female				
53 (74.6%) male				
Possession of driving licenses				
9 (12.7%) licenses for cars and small trucks (basic prerequisite for driving RTV)				
54 (76.1%) basic prerequisite, plus 1 or 2 more licenses for motorcycles, trucks, or trailer				
8 (11.3%) licenses for cars, small trucks, trucks, motorcycles, and trailer				
Place of operation of the driver				
43 (60.6%) urban (>30,000 residents)				
21 (29.6%) suburban (periphery of bigger cities or between 10,000 and 30,000 residents)				
7 (9.9%) rural (<10,000 residents)				

*RTV*, rescue transport vehicle; *SD*, standard deviation.

**Table 2:** Final interrater reliability of two raters (unit of analysis: potentially critical driving situations).

Level	Coding	n	Range	IR
PCDS	Types^b^	216	1–4	.941
PCDS	Road class^a^	216	1–4	.958
PCDS	Size^a^	216	1–3	.959
PCDS	Road condition^b^	216	1–6	.962
PCDS	Weather condition^b^	216	1–7	.837
PCDS	Lightning condition^a^	216	1–4	1.00
PCDS	Reactions to incident^b^	216	Multiple coding	.712
PCDS	Incident type^b^	209	1–9	.919
PCDS	Traffic density^a^	209	1–6	.804
PCDS	Cross traffic^a^	129	1–5	.829
PCDS	Oncoming traffic^a^	93	1–6	.649
PCDS	Traffic in driving direction^b^	75	1–2	.663
Video	Driving style^a^	15	1–3	.926

*n*, number of observed PCDS; *PCDS*, potentially critical driving

situations; *IR*, interrater reliability depending on the data

measurement scale: a=ordinal/interval data with intraclass

correlation (ICC(3,1)), b=nominal data, Cohen’s kappa.

**Table 3:** Environmental ground, weather and light conditions for all incident types (unit of analysis: potentially critical driving situations).

PCDS type	χ^2^ (V)	Right of way (n = 1,177)	Crosswalks (n = 76)	Overtaking (n = 619)	Other (n = 176)	Overall (n = 2,048)
Ground conditions (overall χ^2^ = 16.7; P = .05; Cramer’s V = .052)						
dry	6.8 (.06)	797 (67.7%)	52 (68.4%)	382 (61.7%)	117 (66.5%)	1,348 (65.8%)
humid	2.9 (.04)	241 (20.5%)	20 (26.3%)	144 (23.3%)	39 (22.2%)	444 (21.7%)
wet	10.8* (.07)	125 (10.6%)	3 (3.9%)	90 (14.5%)	19 (10.8%)	237 (11.6%)
snowy	2.6 (.04)	14 (1.2%)	1 (1.3%)	3 (0.5%)	1 (0.6%)	19 (0.9%)
Weather conditions (overall χ^2^ = 19.0, P = .03, Cramer’s V = .056)						
dry	8.9* (.07)	1,005 (85.4%)	66 (86.8%)	496 (80.1%)	149 (84.7%)	1,716 (83.8%)
light rain	4.0 (.04)	152 (12.9%)	9 (11.8%)	100 (16.2%)	23(13.1%)	284 (13.9%)
heavy rain	13.0** (.08)	12 (1.0%)	1 (1.3%)	21 (3.3%)	3 (1.7%)	37 (1.8%)
snow	1.4 (.03)	8 (0.7%)	0 (0%)	2 (0.3%)	1 (0.6%)	11 (0.5%)
Light conditions (overall χ^2^ = 61.2, P < .001, Cramer’s V = .100)						
normal daylight	9.6* (.07)	584 (49.6%)	47 (61.8%)	318 (51.4%)	105 (59.7%)	1,054 (51.5%)
limited daylight	23.1** (.11)	184 (15.6%)	10 (13.2%)	151 (24.4%)	28 (15.9%)	373 (18.2%)
dusk or dawn	6.3 (.06)	65 (5.5%)	2 (2.6%)	50 (8.1%)	11 (6.3%)	128 (6.3%)
darkness	41.8** (.14)	344 (29.2%)	17 (22.4%)	100 (16.2%)	32 (18.2%)	493 (24.1%)

n=number of observed PCDS; values in brackets show the percentage over PCDS type; χ^2^=chi-square with * p ≤ .05, ** p ≤ .01 and V=Cramer’s V (in brackets): statistical different distribution across PCDS between the chosen category of condition compared to the other categories in the respective condition area combined; not observed: icy and heavily soiled ground conditions as well as foggy, hail and windy/stormy weather conditions.

*PCDS*, potentially critical driving situations.

**Table 4:** Reactions to PCDS by ambulance drivers depending on the driving style and PCDS types (unit of analysis: PCDS)

PCDS	Driving Style	χ^2^ (V)	Reactions to PCDS						
			no reaction	swerving	braking	swerving and braking	stopping	stopping acceleration	mixed (swerving, braking, stopping, and/or turning)
Right of way (n = 1,177)	**χ** **^2^** ** (V)**	52.9** (.15)	18.1** (.12)	18.0** (.12)	15.9** (.12)	5.9 (.07)	2.0 (.04)	0.2 (.01)	0.4 (.02)
	defensive driving style (n = 59)	5.1 (.07)	15 (25.4%)	0 (0%)	41 (69.5%)	3 (5.1%)	0 (0%)	0 (0%)	0 (0%)
	normal driving style (n = 963)	35.4** (.17)	195 (20.2%)	14 (1.5%)	620 (64.4%)	118 (12.3%)	9 (0.9%)	1 (0.1%)	6 (0.6%)
	rapid driving style (n = 155)	47.9** (.20)	55 (35.5%)	10 (6.5%)	75 (48.4%)	11 (7.1%)	3 (1.9%)	0 (0%)	1 (0.6%)
Crosswalks (n = 76)	**χ** **^2^** ** (V)**	2.2 (.12)	2.1 (.16)	-	1.4 (.14)	0.5 (.08)	-	-	-
	defensive driving style (n = 7)	1.7. (.15)	6 (85.7%)	0 (0%)	1 (14.3%)	0 (0%)	0 (0%)	0 (0%)	0 (0%)
	normal driving style (n = 65)	2.1 (.17)	39 (60.0%)	0 (0%)	23 (35.4%)	3 (4.6%)	0 (0%)	0 (0%)	0 (0%)
	rapid driving style (n= 4)	0.3 (.07)	3 (75.0%)	0 (0%)	1 (25.0%)	0 (0%)	0 (0%)	0 (0%)	0 (0%)
Overtaking (n = 619)	**χ** **^2^** ** (V)**	29.1** (.15)	4.7 (.09)	22.7** (.19)	2.2 (.06)	13.1** (.15)	0.2 (.02)	-	0.4 (.03)
	defensive driving style (n = 24)	4.9 (.09)	0 (0%)	11 (45.8%)	0 (0%)	13 (54.2%)	0 (0%)	0 (0%)	0 (0%)
	normal driving style (n = 517)	25.5** (.20)	23 (4.4%)	141 (27.3%)	43 (8.3%)	305 (59.0%)	1 (0.2%)	0 (0%)	4 (0.8%)
	rapid driving style (n = 78)	23.0** (.19)	0 (0%)	41 (52.6%)	7 (9.0%)	29 (37.2%)	0 (0%)	0 (0%)	1 (1.3%)
Other (n = 176)	**χ** **^2^** ** (V)**	51.7** (.38)	6.5* (.19)	0.9 (.07)	4.2 (.16)	4.2 (.16)	4.8 (.17)	9.4** (.23)	12.8** (.27)
	defensive driving style (n = 16)	26.5** (.39)	0 (0%)	0 (0%)	7 (43.8%)	1 (6.3%)	0 (0%)	0 (0%)	8 (50.0%)
	normal driving style (n = 143)	20.8* (.34)	11 (7.7%)	4 (2.8%)	62 (43.4%)	12 (8.4%)	33 (23.1%)	0 (0%)	21 (14.7%)
	rapid driving style (n = 17)	26.2** (.39)	4 (23.5%)	0 (0%)	3 (17.6%)	4 (23.5%)	3 (17.6%)	1 (5.9%)	2 (11.8%)
Overall (N = 2,048)	**χ** **^2^** ** (V)**	101.3** (.16)	11.9** (.08)	26.1** (.11)	10.3** (.07)	13.1** (.08)	2.8 (.04)	2.6 (.04)	16.2** (.09)
	defensive driving style (n = 106)	51.4** (.16)	21 (19.8%)	11 (10.4%)	49 (46.2%)	17 (16.0%)	0 (0%)	0 (0%)	8 (7.5%)
	normal driving style (n = 1,688)	51.1** (.16)	268 (15.9%)	159 (9.4%)	748 (44.3%)	438 (25.9%)	43 (2.5%)	1 (0.1%)	31 (1.8%)
	rapid driving style (n = 254)	49.7** (.16)	62 (24.4%)	51 (20.0%)	86 (33.8%)	44 (17.3%)	6 (2.4%)	1 (0.4%)	4 (1.6%)

n=number of observed PCDS, % in brackets = percentage over the driving styles; χ^2^=chi-square with * p ≤ .05, ** p ≤ .01 and V=Cramer’s V (in brackets): χ^2^ in column = statistical differences between the chosen driving style compared to the other two driving styles combined over the reactions to PCDS; χ^2^ in rows = statistical differences between the respective reaction against all other reactions combined over the driving style; χ^2^ in italics report the overall result for 3*7 crosstabs.

*PCDS*, potentially critical driving situations.
