# Supplementary material for: Potentially Critical Driving Situations During “Blue-light” Driving: A Video Analysis
Source: West J Emerg Med. 2023 Jan 3;24(2):348–58. doi: 10.5811/westjem.2022.8.56114 (PMC10047724; doi:10.5811/westjem.2022.8.56114)
Supplement: Supplementary file 1 [file wjem-24-348-s001.pdf]

Supplemental File A: coding protocol for Video analyses (German version)

Kodierungshinweise zu den Videobeurteilungen

Sondersignalnutzung: Zeiten kodieren, wenn nur Teil der Fahrt mit Sondersignal

Fahrweise

1	2	3
defensiv	normal	rasant

Subjektive Einschätzung der Fahrweise aufgrund von Beschleunigung nach Kreuzungen, Abstand halten, Geschwindigkeitsüberschreitungen, abruptem Lenkverhalten

Bodenverhältnisse

1	2	3	4	5	6
trocken	feucht	nass	Eis	Schnee	starke Verschmutzung

Witterungsverhältnisse

1	2	3	4	5	6	7
trocken	Nebel	leichter Regen	starker Regen	Schnee	Hagel	starker Wind/Sturm

Leichter Regen: Scheibenwischer gehen bis zu 20-mal pro Minute
Starker Regen: häufigeres Wischen als 20-mal pro Minute

Lichtverhältnisse

1	2	3	4
normales Tageslicht	eingeschränktes Tageslicht (trübes Wetter)	Abend- oder Morgendämmerung	Dunkelheit

Eingeschränktes Tageslicht: mehr als 70% der Autos fahren mit Licht

Reaktion auf Ereignis

1	2	3	4	5	6	7
keine Reaktion/ gleichmäßiges Weiterfahren	Ausweichen	Bremsen ohne Stillstand	Anhalten/ bis zum Stillstand abbremsen	Beschleunigen	Beschleuni-gungsvorgang unterbrechen	wenden/ geplante Fahrtroute verändern

Bemerkungen:
Unterscheidung zwischen ausweichen und wenden:

> *Wenn der geplante Weg weiter gefahren wird, ist „Ausweichen“ anzukreuzen (z.B.: RTW setzt ein Stück zurück, da er nicht weiter kommt und fährt an einer anderen Seite der Fahrzeuge vorbei).*
> *Wenn ein anderer als geplanter Weg gefahren wird, ist „wenden“ anzukreuzen (z.B. RTW setzt zurück und fährt in eine andere Straße).*

Es können mehrere Kategorien angekreuzt werden. Zum Beispiel „Bremsen“ und „Ausweichen“. „Keine Reaktion“ kann aber nicht mit anderen Kategorien zusammen angekreuzt werden

Supplemental File A: coding protocol for Video analyses (German version)

1. Vorfahrtregelungen an Kreuzungen (auch T-Kreuzungen), Einmündungen und Kreisverkehr

Bemerkungen: Zu kodieren sind alle Verkehrssituationen, in denen mit oder ohne Ampel Vorfahrt gewährt werden muss oder trotz eigener Vorfahrt eine Reaktion erfolgen muss. Zwei Ampeln direkt hintereinander werden jeweils als einzelnes Ereignis erfasst.

Straßentyp

1 Fußgängerzonen
2 städtische Straßen (bis max. 70km/h)
3 Landstraßen, Überortsstraßen (bis max. 100km/h)
4 Autobahn, Schnellstraßen (über 100km/h)

Art der Kreuzung

1 rote Ampel
2 gelbe Ampel
3 (Halt!) Vorfahrt gewähren (Schilder 205, 206)

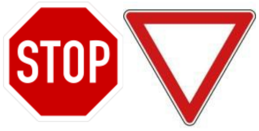

4 Vorfahrt gewähren ohne Beschilderung (rechts vor links, Ausfahrten, etc.)
5 Einsatzfahrzeug hat Vorfahrt (grüne Ampel/entsprechende Beschilderung/ Einmündungen/etc.) wenn Fahrtveränderung erzwungen wird (bremsen, ausweichen)
6 Kreisverkehr

Bemerkungen: die kritischere Art zählt (wenn Ampel von gelb auf rot schaltet → rot kodieren)

Größe der Kreuzung/Anzahl der Fahrstreifen in Fahrtrichtung

1 ein Fahrstreifen
2 zwei Fahrstreifen
3 drei oder mehr Fahrstreifen

Verkehrsdichte (die letzten ca. 30m Innerorts/ca. 60m Außerorts vor der Kreuzung)

1 keine Fahrzeuge
2 vereinzelte Fahrzeuge (bis zu drei Fahrzeuge pro Fahrstreifen/im Kreisverkehr), keine Probleme durchzufahren
3 vereinzelte Fahrzeuge (bis zu drei Fahrzeuge pro Fahrstreifen /im Kreisverkehr), Fahrweg blockiert (auch zeitlich teilweise)
4 dichter Verkehr (über 3 Fahrzeuge pro Fahrstreifen /im Kreisverkehr), keine Probleme durchzufahren
5 dichter Verkehr (über 3 Fahrzeuge pro Fahrstreifen /im Kreisverkehr), Fahrweg blockiert (auch zeitlich teilweise)

Bemerkungen: der freiere Fahrstreifen wird gezählt; Im Kreisverkehr zählt die Anzahl, an der vor und innerhalb des Kreisverkehrs vorbei gefahren werden muss

Querverkehr sowie Fußgänger beim Abbiegen

1 keine Verkehrsteilnehmer
2 stehende Verkehrsteilnehmer, Einsatzfahrzeug kann durchfahren
3 zunächst fahrend/gehend, dann aber Einsatzfahrzeug wahrnehmende Verkehrsteilnehmer (dann stehend)
4 fahrende/gehende Verkehrsteilnehmer, die Einsatzfahrzeug nicht oder zu spät wahrnehmen

Bemerkung: Jeglicher Verkehr, der die Fahrtroute behindern könnte, soll hier erfasst werden. Das kritischere wird kodiert. Wenn alle stehen und einer fährt, wird es als fahrend kodiert.

Gegenverkehr bei Linksabbiegen bzw. wenn auf Gegenspur ausgewichen wird

1 keine Verkehrsteilnehmer
2 stehende Verkehrsteilnehmer, Einsatzfahrzeug kann durchfahren
3 zunächst fahrend/gehend, dann aber Einsatzfahrzeug wahrnehmende Verkehrsteilnehmer (dann stehend)
4 fahrende/gehende Verkehrsteilnehmer, die Einsatzfahrzeug nicht oder zu spät wahrnehmen

Bemerkung: Gegenverkehr nur relevant, wenn links abgebogen wird, Querverkehr soll immer beachtet werden. Das kritischere wird kodiert. Wenn alle stehen und einer fährt, wird es als fahrend kodiert.

2. <u>Fußgängerüberwege und Fußgängerampeln</u>

Zu kodieren sind alle Verkehrssituationen, in denen das Einsatzfahrzeug an einen Fußgängerüberweg kommt, unabhängig davon, ob eine Person diesen benutzen möchte oder nicht (denn dies muss zumindest vom Einsatzfahrer beachtet werden. Weiterhin sollen Fußgängerampeln kodiert werden, wenn diese rot oder gelb sind oder wenn bei grünem Licht eine Reaktion erforderlich ist.

Straßentyp

1 Fußgängerzonen
2 städtische Straßen (bis max. 70km/h)
3 Landstraßen, Überortsstraßen (bis max. 100km/h)

Art des Überweges

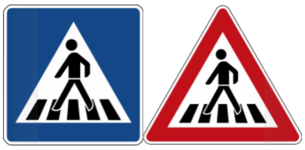

1 rote Ampel
2 gelbe Ampel
3 grüne Ampel, wenn Reaktion erforderlich
4 Fußgängerüberweg (Schilder 350-10, 134-10)
Bemerkungen: das kritischere zählt (wenn Ampel von gelb auf rot schaltet → rot kodieren)

Größe: Anzahl der Fahrstreifen in Fahrtrichtung vor der Ampel/dem Überweg

1 ein Fahrstreifen
2 zwei Fahrstreifen
3 drei oder mehr Fahrstreifen

Verkehrsdichte (auf Überweg bzw. 5m davor und dahinter)

1 keine Fahrzeuge
2 vereinzelte Fahrzeuge (bis zu drei Fahrzeuge pro Fahrstreifen/im Kreisverkehr), keine Probleme durchzufahren
3 vereinzelte Fahrzeuge (bis zu drei Fahrzeuge pro Fahrstreifen /im Kreisverkehr), Fahrweg blockiert (auch zeitlich teilweise)
4 dichter Verkehr (über 3 Fahrzeuge pro Fahrstreifen /im Kreisverkehr), keine Probleme durchzufahren
5 dichter Verkehr (über 3 Fahrzeuge pro Fahrstreifen /im Kreisverkehr), Fahrweg blockiert (auch zeitlich teilweise)

Querverkehr: Fußgänger

1 keine Fußgänger
2 vereinzelte Fußgänger (bis zu drei), die Platz machen und RTW wahrgenommen haben
3 vereinzelte Fußgänger (bis zu drei), Fahrtweg blockiert (auch zeitlich teilweise)
4 viele Fußgänger (über 3), die Platz machen und RTW wahrgenommen haben
5 viele Fußgänger (über 3), Fahrtweg blockiert (auch zeitlich teilweise)
Bemerkungen: Zu Fußgängern zählen hier auch Radfahrer und jegliche Zusatzgeräte (Skateboards, Kinderwägen, Pferde…), wenn der Überweg benutzt werden soll

Gegenverkehr wenn relevant (bspw. Ausweichen auf Gegenspur, da Fußgänger auf Straße)

1 keine Verkehrsteilnehmer
2 stehende Verkehrsteilnehmer, Einsatzfahrzeug kann durchfahren
3 zunächst fahrend/gehend, dann aber Einsatzfahrzeug wahrnehmende Verkehrsteilnehmer (dann stehend)
4 fahrende/gehende Verkehrsteilnehmer, die Einsatzfahrzeug nicht oder zu spät wahrnehmen
Bemerkung: Das kritischere wird kodiert. Wenn alle stehen und einer fährt, wird es als fahrend kodiert.

3. **<u>Überholen</u>**

Bemerkung: Auch das nicht vollständige Verlassen der Fahrbahn in Fahrtrichtung beim Überholen ist als solches Ereignis zu kodieren. Wenn RTW auf Fahrstreifen zurück wechselt und neuen Überholvorgang beginnt, neues Ereignis kodieren; bleibt er auf der Gegenfahrbahn und überholt auch in Abständen, bleibt es ein Ereignis. Überholvorgänge können auch passieren, ohne dass in den Gegenverkehr gefahren wird, wenn beispielweise Stau auftritt oder zwischen den Fahrzeugen überholt wird. Nutzt das Einsatzfahrzeug eine Sonderspur zum Überholen (bspw. Eine Busspur), so wird dieses Ereignis nicht kodiert, es sei denn, eine Reaktion ist erforderlich oder es erscheint kritisch (bei Einfahren/Ausfahren von solchen Spuren kann es zu einem sonstigen kritischen Ereignis kommen (siehe 4. Art 8).

Straßentyp

1 Fußgängerzonen
2 städtische Straßen (bis max. 70km/h)
3 Landstraßen, Überortsstraßen (bis max. 100km/h)
4 Autobahn, Schnellstraßen (über 100km/h)

Art des Überholens:

1 Gerade, überschaubar
2 Gerade, unübersichtlich
3 Kurve, überschaubar
4 Kurve, unübersichtlich
5 Stausituation

Größe: Anzahl der Fahrstreifen in Fahrtrichtung

1 ein Fahrstreifen
2 zwei Fahrstreifen
3 drei oder mehr Fahrstreifen

Verkehrsdichte in Fahrtrichtung

1 vereinzelte Fahrzeuge (bis zu drei Fahrzeuge)
2 Fahrzeugkolonne (vier bis zehn Fahrzeuge)
3 Fahrzeugkolonne (ab zehn Fahrzeuge)

Querverkehr

Nur kodieren, wenn relevant, dann Orientierung an Kreuzungssituationen

Gegenverkehr (in bis zu 50m Abstand Innerorts/100m Außerorts)

1 kein Gegenverkehr
2 Gegenverkehr steht
3 Gegenverkehr fährt
4 rechts überholen, daher kein Gegenverkehr
5 überholen zwischen den Spuren, daher kein Gegenverkehr
6 nicht relevant, da Gegenverkehr baulich getrennt ist

Verkehr in Fahrtrichtung

1 Fahrzeuge stehen
2 rollender Verkehr

Bemerkung: das kritischere wird kodiert, wenn alle stehen, aber einer fährt, rollender Verkehr.

4. Sonstiges (Eingreifen erforderlich)

Bemerkungen: hier sollen alle Ereignisse kodiert werden, die in irgendeiner Weise eine erhöhte Aufmerksamkeit erfordern oder zu Unfällen führen können. Einige Arten sind bereits vorgegeben können aber mit sonstigen weiter definiert werden. Hier sollen auch alle Unterkategorien genutzt werden, die sich auf das Ereignis anpassen lassen, sonst soll es frei bleiben (z.B. Gegenverkehr, wenn er relevant ist, Querverkehr)
Eigenangaben sollen ähnlich kodiert werden, wenn sie nicht bereits in anderen Kategorien kodiert wurden.

Straßentyp

1 Fußgängerzonen
2 städtische Straßen (bis max. 70km/h)
3 Landstraßen, Überortsstraßen (bis max. 100km/h)
4 Autobahn, Schnellstraßen (über 100km/h)

Art des kritischen Ereignisses

1 Behinderung durch andere Verkehrsteilnehmer
2 Fahrt gegen vorgeschriebene Fahrtrichtung (Einbahnstraßen, Kreisverkehr, …)
3 andere Sondersignalfahrzeuge
4 enge Stelle
5 Tiere:___________________(angeben welches)
6 Unfall
7 „Spurwechsel“ auf nicht vorhandene bzw. besondere Fahrbahn wenn besondere Aufmerksamkeit erforderlich (bspw. Busspur, Straßenbahn, Grasnarbe, Fußgängerweg)
8 verfahren/zu weit gefahren
9 Sonstige:____________________________(angeben welche Art)

Größe: Anzahl der Fahrstreifen in Fahrtrichtung

1 ein Fahrstreifen
2 zwei Fahrstreifen
3 drei oder mehr Fahrstreifen

Verkehrsdichte in Fahrtrichtung

1 keine Fahrzeuge/Fußgänger/Verkehrsteilnehmer
2 vereinzelte Verkehrsteilnehmer (bis zu drei), keine Probleme durchzufahren
3 vereinzelte Verkehrsteilnehmer (bis zu drei), Fahrweg blockiert (auch zeitlich teilweise)
4 dichter Verkehr (über 3 Verkehrsteilnehmer), keine Probleme durchzufahren
5 dichter Verkehr (über 3 Verkehrsteilnehmer), Fahrweg blockiert (auch zeitlich teilweise)

Querverkehr

1 keine Verkehrsteilnehmer
2 stehende Verkehrsteilnehmer, Einsatzfahrzeug kann durchfahren
3 zunächst fahrend/gehend, dann aber Einsatzfahrzeug wahrnehmende Verkehrsteilnehmer (dann stehend)
4 fahrende/gehende Verkehrsteilnehmer, die Einsatzfahrzeug nicht oder zu spät wahrnehmen

Bemerkung: Jeglicher Verkehr, der die Fahrtroute behindern könnte, soll hier erfasst werden. Das kritischere wird kodiert. Wenn alle stehen und einer fährt, wird es als fahrend kodiert.

Gegenverkehr

1 keine Verkehrsteilnehmer
2 stehende Verkehrsteilnehmer, Einsatzfahrzeug kann durchfahren
3 zunächst fahrend/gehend, dann aber Einsatzfahrzeug wahrnehmende Verkehrsteilnehmer (dann stehend)
4 fahrende/gehende Verkehrsteilnehmer, die Einsatzfahrzeug nicht oder zu spät wahrnehmen

Bemerkung: Gegenverkehr nur relevant, wenn links abgebogen wird, Querverkehr soll immer beachtet werden. Das kritischere wird kodiert. Wenn alle stehen und einer fährt, wird es als fahrend kodiert
