# Supplementary material for: Potentially Critical Driving Situations During “Blue-light” Driving: A Video Analysis
Source: West J Emerg Med. 2023 Jan 3;24(2):348–58. doi: 10.5811/westjem.2022.8.56114 (PMC10047724; doi:10.5811/westjem.2022.8.56114)
Supplement: Supplementary file 2 [file wjem-24-348-s002.pdf]

Supplemental File B: coding protocol for Video analyses (English version)

Coding Protocol for Video analyses

Driving with blue light and sirens: code times of each incident

Driving style

1	2	3
defensive	normal	rapid

Subjective assessment of the driving style due to acceleration after intersections, keeping distances, exceeding speed limits and sharp steering behavior

Ground Conditions

1	2	3	4	5	6
dry	humid	wet	icy	snowy	heavy dirt

Weather Conditions

1	2	3	4	5	6	7
dry	foggy	light rain	heavy rain	snow	hail	windy/stormy

Light rain: wipers go slower than 20 per minute
Heavy rain: wipers go faster than 20 per minute

Light Conditions

1	2	3	4
normal daylight	limited daylight (dull weather)	dusk or dawn	darkness

Limited daylight: more than 70% of the vehicles driving with lights on

Reaction to incident

1	2	3	4	5	6	7
no reaction/ consistent driving	swerving	braking without stopping	stopping/ braking to a halt	accelerate	stopping acceleration	turning/using an alternate route

Comments:
Differentiation between swerving and turning:

> *When planed route is still followed "swerving" needs to be marked (e.g., ambulance drives back a bit due to a blocked way and then passes vehicles on another side).*
>
> *When another than planed route is used "turning" needs to be marked (e.g., ambulance drives back a bit due to a blocked way and then uses another street).*

It is possible to mark more than one reaction. For example "braking" and "swerving". However, "no reaction" cannot be mixed with other reactions.

Supplemental File B: coding protocol for Video analyses (English version)

1. **Right of Way (all kinds of crossings, junctions, side roads and roundabouts)**

   Comments: Code all traffic situations in which the ambulance needs to give way to another road user no matter if there is a light, sign or none of them. Also code situations in which the ambulance has right of way but needs to react to other road users (e.g., a traffic jam in front of a green light). Two traffic lights in series are coded as two single incidents.

Road Class

1 pedestrian area
2 urban street (up to a maximum of 70km/h)
3 rural street (up to a maximum of 100km/h)
4 autobahn (usually more than 100km/h)

Incident Type

1 red light
2 yellow light
3 stop or yield sign (German road sign nr. 205 and 206)

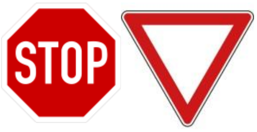

4 give way without a traffic sign (priority-to-the-right-rule, exits, etc.)
5 ambulance has right of way (green light/appropriate signage/junctions/etc.) with enforced change of driving (braking, swerving)
6 roundabout

Comments: the more critical type needs to be coded if two types fit the incident (e.g., light changes from yellow to red → "red light" needs to be coded)

Size: number of lanes in driving direction

1 one lane
2 two lanes
3 three or more lanes

Traffic Density (about the last 30m (urban)/60m (rural) before the crossing)

1 no vehicles
2 few vehicles (up to three per lane) without problems to pass
3 few vehicles (up to three per lane) with (partially) blocked road
4 heavy traffic (more than three vehicles per lane) without problems to pass
5 heavy traffic (more than three vehicles per lane) with (partially) blocked road

Comments: the lane with the fewest vehicles hast o be counted; in roundabouts the number of vehicles count that need to be passed right before and inside the roundabout

Cross Traffic and pedestrians when turning

1 no road users
2 staying road users the ambulance can pass
3 initially driving/moving road users then staying and letting ambulance pass
4 driving/moving road users that notice ambulance late or not at all

Comments: Any traffic that could hinder the driving route of the ambulance needs to be coded. The more critical rating needs to be coded (e.g., all traffic users but one are staying → code as driving).

Oncoming Traffic when turning left or swerving into oncoming traffic lane

1 no road users
2 staying road users the ambulance can pass
3 initially driving/moving road users then staying and letting ambulance pass
4 driving/moving road users that notice ambulance late or not at all

Comments: Oncoming traffic is just relevant when turning left, Cross traffic should be coded always. The more critical rating needs to be coded (e.g., all traffic users but one are staying → code as driving).

2. Pedestrian Crosswalks and Pedestrian lights

Comments: Code all traffic situations where the ambulance gets to a pedestrian crossing no matter if a pedestrian wants to cross it (the driver or the ambulance needs to consider the risk in any case). Pedestrian lights always need to be coded if red or yellow and additionally if green but a reaction is necessary.

Road Class

1 pedestrian area
2 urban street (up to a maximum of 70km/h)
3 rural street (up to a maximum of 100km/h)

Incident Type

1 red light
2 yellow light
3 green light, a reaction is necessary
4 pedestrian crossing (German traffic sign nr. 350-10 or 134-10)

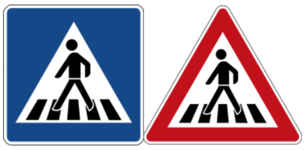

Comments: the more critical type needs to be coded (e.g., light changes from yellow to red → "red light" needs to be coded)

Size: number of lanes in driving direction before the lights/pedestrian crossing

1 one lane
2 two lanes
3 three or more lanes

Traffic Density (on the pedestrian crossing and 5m before and after it)

1 no vehicles
2 few vehicles (up to three per lane) without problems to pass
3 few vehicles (up to three per lane) with (partially) blocked road
4 heavy traffic (more than three vehicles per lane) without problems to pass
5 heavy traffic (more than three vehicles per lane) with (partially) blocked road

Cross Traffic: Pedestrians

1 no pedestrians
2 few pedestrians (up to three) that noticed the ambulance and give way
3 few pedestrians (up to three) with (partially) blocked road
4 many pedestrians (more than three) that noticed the ambulance and give way
5 many pedestrians (more than three) with (partially) blocked road

Comments: Other road users and using additional equipment (cyclists, skateboards, buggies, horses, etc) are count as pedestrians if they want to use the crossing.

Oncoming Traffic when relevant (swerving into oncoming traffic lane due to pedestrians on the sreet)

1 no road users
2 staying road users the ambulance can pass
3 initially driving/moving road users then staying and letting ambulance pass
4 driving/moving road users that notice ambulance late or not at all

Comments: Oncoming traffic is just relevant when turning left, Cross traffic should be coded always. The more critical rating needs to be coded (e.g., all traffic users but one are staying → code as driving).

3. Overtaking

Comments: All overtaking situations need to be coded even if the ambulance does not fully leave the lane. When the ambulance changes back to the actual lane and leaves it again a new overtaking incident needs to be coded. If the ambulance stays on the oncoming traffic lane without changing back to the actual lane just one overtaking incident needs to be coded. Overtaking also can occur without driving on the oncoming traffic lane, for example when overtaking in a traffic jam on the autobahn or between the lanes. If the ambulance uses a special lane like a bus lane the incident will not be coded as an overtaking maneuver, except a reaction is required or it seem critical. Driving on or of such a special lane can be coded as an "other" incident (fourth category, incident type 8).

Road Class

1 pedestrian area
2 urban street (up to a maximum of 70km/h)
3 rural street (up to a maximum of 100km/h)
4 autobahn (usually more than 100km/h)

Incident Type

1 clear straight road
2 unclear straight road
3 clear bend
4 blind bend
5 traffic jam

Size: number of lanes in driving direction

1 one lane
2 two lanes
3 three or more lanes

Traffic Density in driving direction

1 few vehicles (up to three vehicles)
2 small convoy (four up to nine vehicles)
3 large convoy (ten or more vehicles)

Cross Traffic

Comments: just coding when relevant; then code like category 1.

Oncoming Traffic (until about a distance of 50m (urban)/100m (rural))

1 no oncoming traffic
2 oncoming traffic is staying
3 oncoming traffic is driving
4 overtaking on the right-hand side (no oncoming traffic)
5 overtaking between the traffic lanes (no oncoming traffic)
6 not relevant due to a constructional separation to the oncoming traffic

Traffic in driving direction

1 staying vehicles
2 driving vehicles

Comments: The more critical rating needs to be coded (e.g., all traffic users but one are staying → code as driving).

4. <u>Other Critical Incidents (intervention needed)</u>

Comments: all incidents need to be coded that require in any kind an increased attention or could lead to an accident. Some incident types are defined already, but any other incidents can be coded in this category and then must be named. Other sub-categories of the first three categories can but do not have to be used adapted in this category (e.g. oncoming or cross traffic when relevant). Here should also be coded all critical incidents that were named by the drivers at the end of the shift if not already coded somewhere else.

Road Class

1 pedestrian area
2 urban street (up to a maximum of 70km/h)
3 rural street (up to a maximum of 100km/h)
4 autobahn (usually more than 100km/h)

Incident Type

1 hindrance by other road users
2 driving wrong way (one-way-street, roundabouts, etc)
3 other blue light driving vehicle
4 narrow road
5 animals: _________________ (name what animal)
6 having an accident
7 lane change on special roads (like bus or tram lanes, turf or walkways)
8 lose one's way or turning
9 others: __________________________ (name what kind)

Size: number of lanes in driving direction

1 one lane
2 two lanes
3 three or more lanes

Traffic Density

1 no vehicles
2 few vehicles (up to three per lane) without problems to pass
3 few vehicles (up to three per lane) with (partially) blocked road
4 heavy traffic (more than three vehicles per lane) without problems to pass
5 heavy traffic (more than three vehicles per lane) with (partially) blocked road

Comments: the lane with the fewest vehicles hast o be counted

Cross Traffic and pedestrians when turning

1 no road users
2 staying road users the ambulance can pass
3 initially driving/moving road users then staying and letting ambulance pass
4 driving/moving road users that notice ambulance late or not at all

Comments: Any traffic that could hinder the driving route of the ambulance needs to be coded. The more critical rating needs to be coded (e.g., all traffic users but one are staying → code as driving).

Oncoming Traffic when turning left or swerving into oncoming traffic lane

1 no road users
2 staying road users the ambulance can pass
3 initially driving/moving road users then staying and letting ambulance pass
4 driving/moving road users that notice ambulance late or not at all

Comments: Oncoming traffic is just relevant when turning left, Cross traffic should be coded always. The more critical rating needs to be coded (e.g., all traffic users but one are staying → code as driving).
