# Supplementary material for: Potentially Critical Driving Situations During “Blue-light” Driving: A Video Analysis
Source: West J Emerg Med. 2023 Jan 3;24(2):348–58. doi: 10.5811/westjem.2022.8.56114 (PMC10047724; doi:10.5811/westjem.2022.8.56114)
Supplement: Supplementary file 3 [file wjem-24-348-s003.pdf]

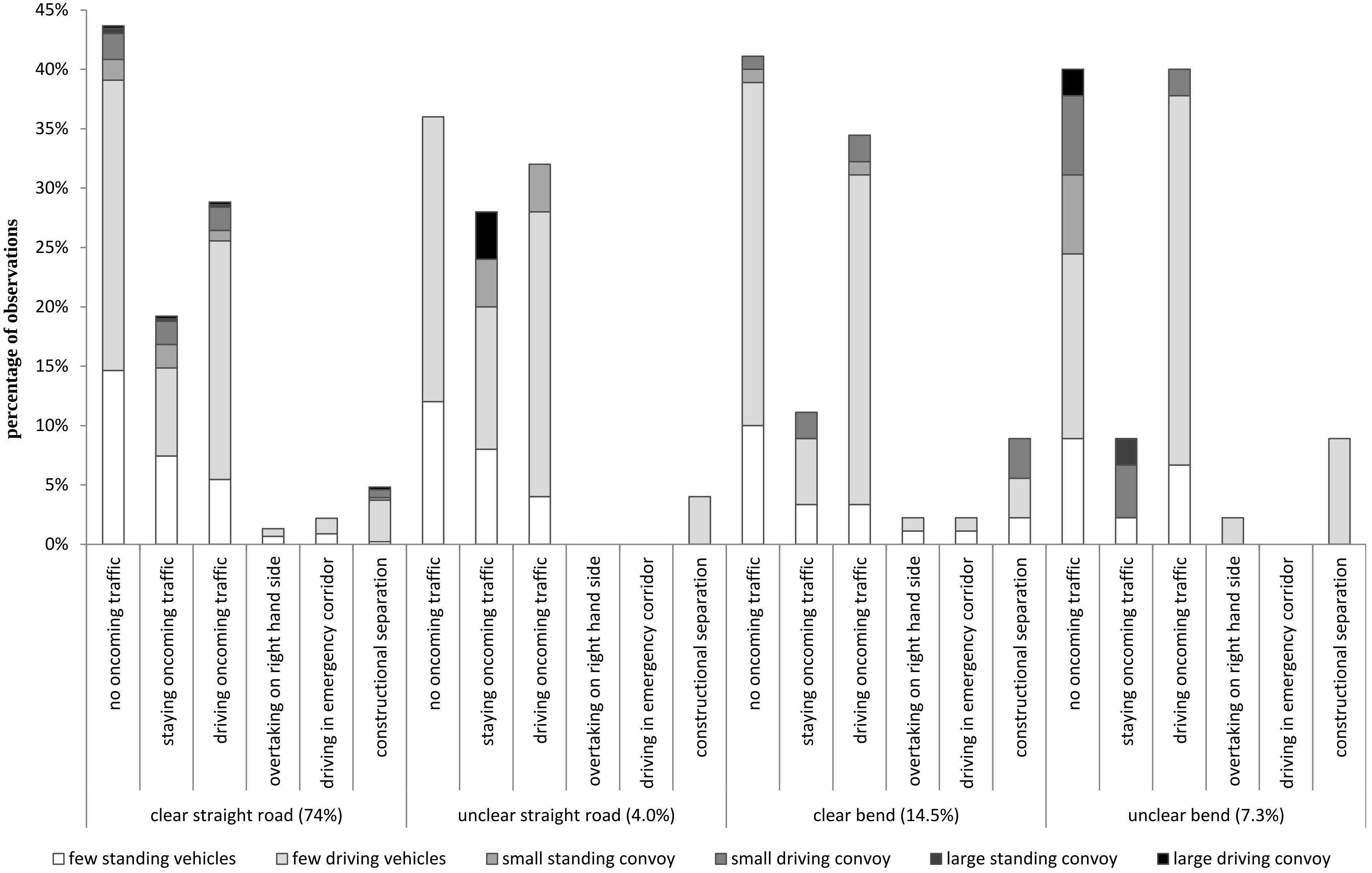

Figure C-1. Combination of oncoming traffic (bars) and traffic in driving direction (legend) for overtaking maneuvers depending on the incident type (horizontal axis). The incident type traffic jam (0.2%) is excluded due to 100% being a large driving convoy with a constructional separation to oncoming traffic.

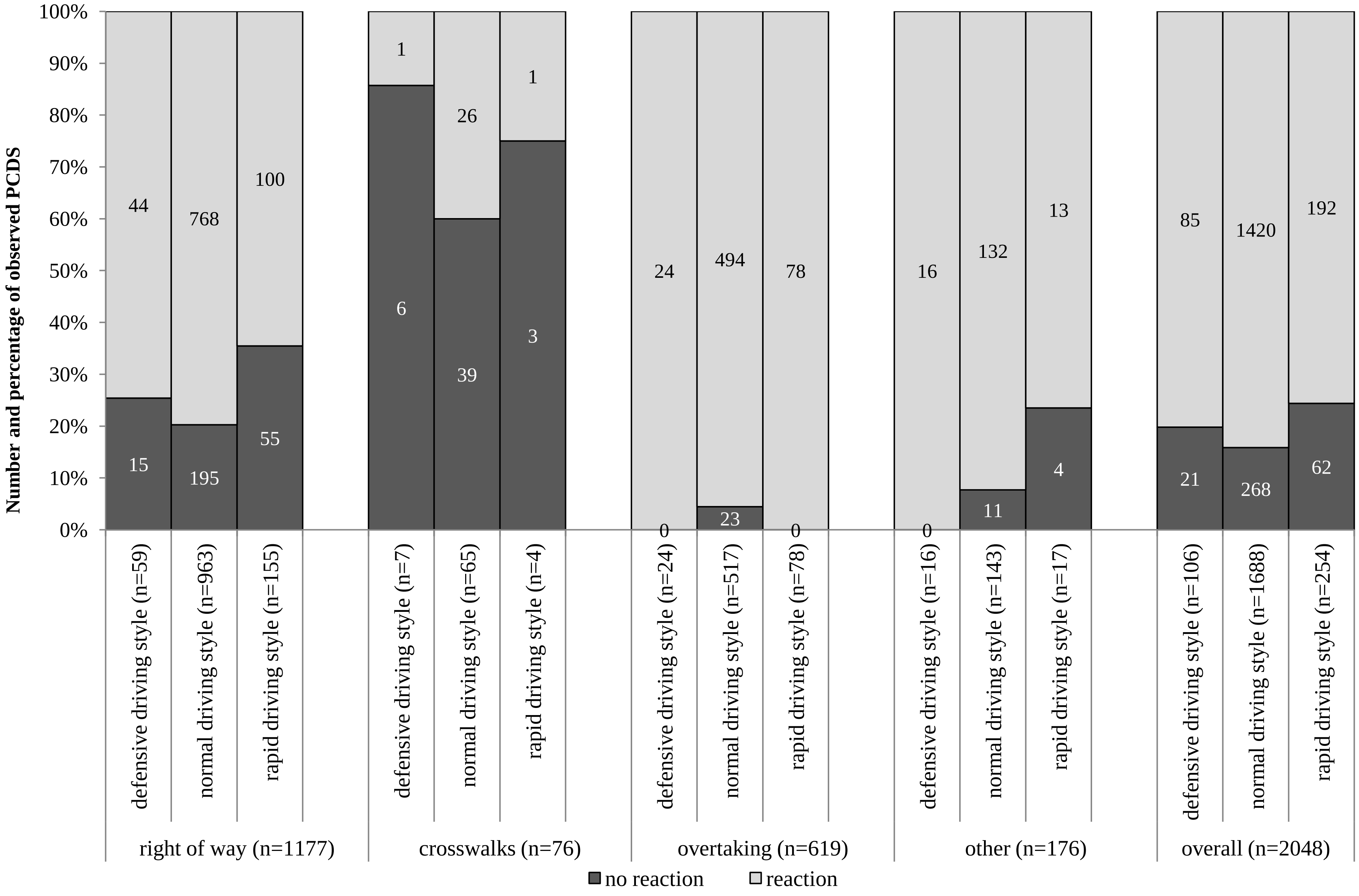

Figure C-2. Occurrence of any reaction to PCDS by incident type and driving style. Reactions include swerving, braking, stopping, acceleration, stopping acceleration, turning or a mix of those.
